# Comparing ventral and dorsal oral mucosal graft urethroplasty in female urethral stricture: a systematic review and meta-analysis

**DOI:** 10.1007/s00345-025-05773-4

**Published:** 2025-06-26

**Authors:** Mazhar Ortac, M. Firat Ozervarli, Rifat Burak Ergul, Arda Tunc Aydinoglu, Mevlut Melih Bicer, Teresa Olsen Ekerhult, Senol Tonyali, Assoc Prof

**Affiliations:** 1 Department of Urology, Istanbul Faculty of Medicine, Istanbul University, Topkapı, Turgut Özal Millet Street, Fatih, 34093 Istanbul, Turkey; 2 Department of Urology, Institute of Clinical Sciences, Sahlgrenska Academy, University of Gothenburg, Gothenburg, Sweden

**Keywords:** Female urethroplasty, Oral mucosal graft, Buccal mucosa, Ventral, Dorsal

## Abstract

**Purpose:**

Reconstructive surgical options have become an alternative for female urethral stricture. Dorsal and ventral methods using oral mucosa grafts have been described, but their superiority over each other has not been evaluated. In this meta-analysis, the outcomes of dorsal and ventral techniques with oral mucosa graft in female urethroplasty have been compared.

**Material and method:**

A systematic search of Pubmed, Scopus and Web of Science databases was performed according to the Preferred Reporting Items For Systematic Review And Meta-Analysis statement. Manuscripts published until February 2025 that reported the use of dorsal or ventral surgical approaches with oral mucosa grafts in female urethroplasty included. Success, determined based on the recurrence rate, was analyzed and compared between both groups. Complications were presented in both groups to evaluate the safety of the surgical technique.

**Results:**

A total of 320 studies were identified, with 25 meeting the inclusion criteria. The meta-analysis, including four comparative cohort studies, showed no significant difference in surgical success between the dorsal (62/69 patients) and ventral (93/103 patients) techniques (OR = 0.84, 95% CI: 0.30–2.36, *p* = 0.74). In the pooled analysis combining both comparative and non-comparative studies, the success rates for the dorsal and ventral techniques were 92.1% (95% CI: 89.1–95.1) and 95.5% (95% CI: 92.8–98.2), respectively, with no significant heterogeneity. A total of 4 cases of stress urinary incontinence complications were reported in the ventral approach, while 2 cases were reported using the dorsal approach. The majority of the studies (21 out of 25) provided Level 4 evidence, with only two randomized controlled trials reaching Level 2.

**Conclusion:**

This meta-analysis confirms that both dorsal and ventral approaches are effective for treating female urethral stricture, with high success rates. There was no statistically significant difference in success rates between the ventral and dorsal approaches. Surgical approach selection should depend on patient factors and surgeon expertise to achieve optimal outcomes.

**Supplementary Information:**

The online version contains supplementary material available at 10.1007/s00345-025-05773-4.

## Introduction

Female urethral strictures are relatively rare, and considerable ambiguity persists regarding their accurate diagnosis and optimal management strategies [1]. Epidemiological data indicate that urethral strictures occur in 4–20% of women diagnosed with bladder outflow obstruction [2–4]. The true prevalence of female urethral stricture (FUS) among women presenting with voiding dysfunction is estimated to range between 0.1% and 1% [5, 6]. Etiologically, the development of female urethral stricture disease may be attributed to factors such as trauma, iatrogenic interventions, inflammatory processes, lichen sclerosus, radiation, or malignant lesions [7].

Although various treatment approaches exist, urethral dilation remains the first-line intervention for FUS [8]. Optical internal urethrotomy is another therapeutic option; however, it carries a risk of sphincteric injury, potentially leading to stress urinary incontinence (SUI) [9]. While these minimally invasive treatments may provide temporary symptom relief, they fail to address the underlying fibrotic mechanism of stenosis, resulting in a high recurrence rate [10]. Despite their low complication rates, meta-analyses have demonstrated poor long-term success with these approaches [11]. Consequently, there has been a growing interest in reconstructive urology, driving the development of various surgical techniques aimed at providing durable outcomes and achieving high success rates in the management of FUS [12].

Over time, various urethroplasty techniques have been developed based on the type of graft or flap utilized, including vaginal, labial/vestibular, buccal, and lingual grafts, as well as vaginal and labial/vestibular flaps [13]. In urethroplasty utilizing oral mucosa (lingual or buccal), either a ventral or dorsal approach may be preferred, depending on the specific anatomical and clinical considerations of the patient. Each approach offers distinct advantages. The ventral approach facilitates surgical preparation and exposure, providing optimal visualization and access to the bladder neck. Furthermore, it is postulated that this approach minimizes the risk of stress incontinence and sexual dysfunction by reducing the potential for damage to the sphincter muscle and neurovascular bundle located dorsaly [14]. Conversely, the dorsal approach offers mechanical stability and enhanced graft vascularization, contributing to improved graft integration. Its anatomical positioning promotes physiological urine flow and reduces the risk of urethrovaginal fistula formation, making it a favorable option in select cases [15].

Despite the widespread adoption of both dorsal and ventral approaches in the surgical management of female urethral stricture, the existing literature lacks a comprehensive comparative analysis of their efficacy and reliability. This gap in knowledge has contributed to ongoing uncertainty in clinical decision-making. To address this issue, we present a meta-analysis that directly compares the success rates and reliability of dorsal and ventral urethroplasty techniques. By synthesizing the available evidence, this study aims to provide evidence-based guidance for selecting the most effective surgical approach in female urethral reconstruction.

## Method

### Design and registration

This systematic review and meta-analysis were conducted in accordance with The Cochrane Handbook for Systematic Reviews of Interventions and the Preferred Reporting Items for Systematic Reviews and Meta-analyses (PRISMA) guidelines [16]. Additionally, the protocol was registered in the International Prospective Register of Systematic Reviews (PROSPERO) database with ID “CRD42025637591”.

### Information sources and search strategy

A comprehensive search was conducted across the following databases: PubMed, Scopus and Web of Science. Different combinations of the following keywords were used to search for articles by title/abstract: “urethroplasty”, “urethral stricture”, “female”, “women”, “buccal”, “lingual”, “ventral”, “dorsal”, “ventral inlay”, “ventral onlay”, “dorsal onlay”. The search was limited to studies published up to February 2025. The search strings are reported in Supplementary Table 1.

Once the identified studies were imported into Rayyan software [17], duplicate publications were detected and subsequently excluded from the analysis.

We also manually searched the reference lists of relevant studies and reviews to identify additional eligible studies.

### Study selection

Two independent reviewers (MFO and RBE) screened the titles and abstracts of all identified studies. Full-text articles were reviewed for studies that met the eligibility criteria. During this process, conflicts or discrepancies were settled by agreement with the supervisor (MO).

The systematic review and meta-analysis were designed using the PICOS framework to define the scope and inclusion criteria for comparing the ventral and dorsal oral mucosa graft (lingual or buccal) methods in female urethroplasty:

### Population (P)

This review includes studies involving female patients undergoing urethroplasty using oral mucosa grafts. All female patients with urethral stricture due to any etiology were included.

### Intervention (I)

The review compares two urethroplasty techniques:

- Ventral urethroplasty technique: İnvolves performing urethroplasty through a ventral incision at the 6 o’clock position of the urethra, with oral mucosal grafts utilized for reconstruction [18].
- Dorsal urethroplasty technique: entails a dorsal incision at the 12 o’clock position, similarly employing oral mucosal grafts for urethral reconstruction [18].

### Comparator (C)

The comparison is made between the two urethroplasty techniques (ventral vs. dorsal) to assess their relative effectiveness, safety, and outcomes.

### Outcome (O)

The outcomes evaluated include surgical success rates (e.g., recurrence of stricture), and complication rates at any time during the follow-up period (e.g., infection, stress incontinence, ).

### Study design (S)

This meta-analysis includes both prospective and retrospective studies, as well as randomized and non-randomized trials. It comprises comparative studies that directly compare ventral and dorsal urethroplasty techniques, as well as case series reporting outcomes of either technique in female patients.

### Inclusion and exclusion criteria

The criterion for inclusion in the study was the clear specification of either the ventral or dorsal surgical technique. Recurrence of urethral stricture after urethroplasty was considered a failure. The definition of recurrence was established as the development of a stricture at any time during the follow-up period that would require surgical intervention, including urethral dilation, internal urethrotomy, or urethroplasty. Therefore, studies with clearly defined recurrence rates were included to analyze the success of the procedure. Reviews, meta-analyses, letters, editorial comments, or conference abstracts, and case series fewer than 10 patients excluded. Studies in languages other than English and animal studies were also excluded from the meta-analysis.

### Data extraction

Data extraction was performed using Excel, where a structured table was created to compile key study characteristics, including author, year of publication, number of participants, patient age, follow-up duration, prior interventions, type of graft used, surgical technique, postoperative evaluation methods, success rates, and reported complications.

Each reviewer (MFO and RBE) independently extracted the data, and the results were subsequently cross-checked to ensure accuracy and eliminate potential errors. No assumptions or simplifications were made during the extraction process. All relevant data were obtained directly from the included studies, and it was not necessary to contact any authors for clarifications or additional information.

### Quality assessment

The level of evidence (LoE) for all included studies was evaluated following the guidelines of the Oxford Centre for Evidence-Based Medicine (2011). The quality assessment of randomized controlled trials (RCTs) was conducted using the Jadad Scale, while comparative nonrandomized studies were assessed with the Newcastle-Ottawa Scale (NOS). For noncomparative studies, the JBI Critical Appraisal Tool was applied to ensure a rigorous evaluation of methodological quality. The JBI tool was used in 21 of the 25 articles included in the review for non-comparative studies (Supplementary Table 2).

### Statistical analysis

The first meta-analysis included four studies with a comparative cohort design involving both dorsal and ventral patient groups. The success of the surgical technique was defined as an “event”. The analyses were performed using RevMan software and applied the Mantel-Haenszel method. Odds Ratio (OR) was used as the effect measure, and the meta- analysis was conducted using a Fixed-Effect Model. Total event counts and estimated OR were calculated with 95% confidence intervals (CI). Heterogeneity was assessed using the chi-squared (Chi²) test and reported with the I² statistic. The second and third meta- analyses compared the success rates of two surgical techniques: Dorsal and Ventral. The studied were divided into two groups based on the surgical techniques, with studies involving both surgical techniques included in both groups. The primary outcome was defined as the proportion of successful cases among the total number of patients. Statistical analyses were conducted using R version 2023.06.1 + 524 and the meta package. The meta-analyses for both techniques were performed separately using the metaprop function, employing a logit transformation (PLN) and the Inverse-Variance Method for pooling. Confidence intervals were calculated using the Clopper-Pearson method. Forest plots were generated to visualize the success rates and their 95% confidence intervals for each technique. The pooled success rates and standard errors for both techniques were extracted, and a Z-test was conducted to compare these rates. The Z-score and p-value were calculated, with a p-value <0.05 considered statistically significant. Additionally, funnel plots were created for each technique to evaluate potential publication bias.

**Fig. 1:**
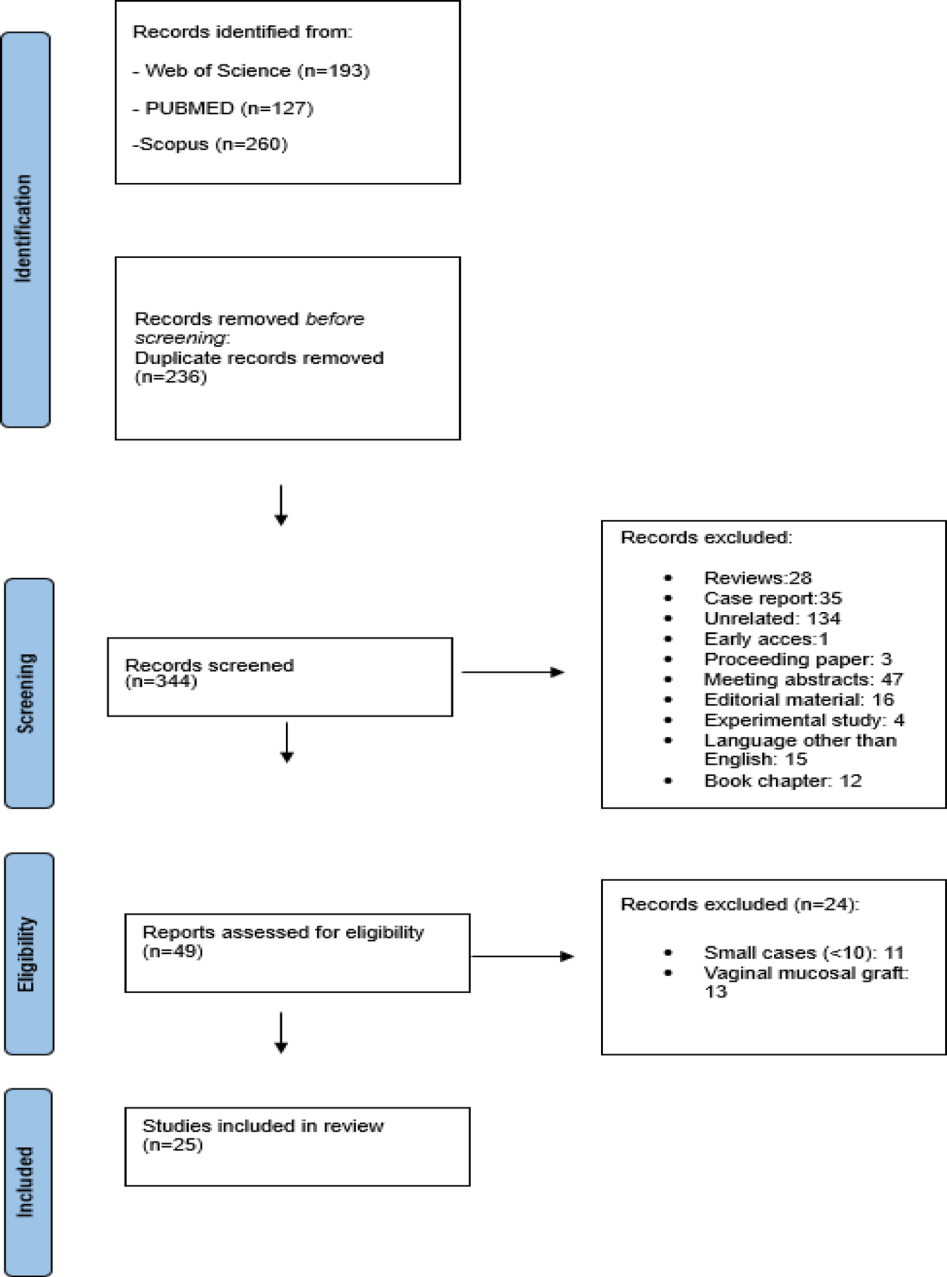
Prisma flowchart adapted for the selection of studies included

## Results

A total of 320 studies were initially identified (Fig. 1). After excluding studies based on various criteria 25 full-text articles met the inclusion criteria and were included in the final analysis. Of the 25 studies included in this analysis, 7 were prospective and 18 were retrospective in design (Supplementary Table 2). The mean/median follow-up durations ranged from 6 to 66 months, and the reported mean/median patient ages varied between 39 and 58 years. Twelve studies utilized the dorsal approach, nine employed the ventral approach, and four comparative studies evaluated both techniques directly. Regarding graft material, only two studies reported the use of lingual mucosa, while the remaining studies used buccal mucosa (Table 1).

**Fig. 2:**
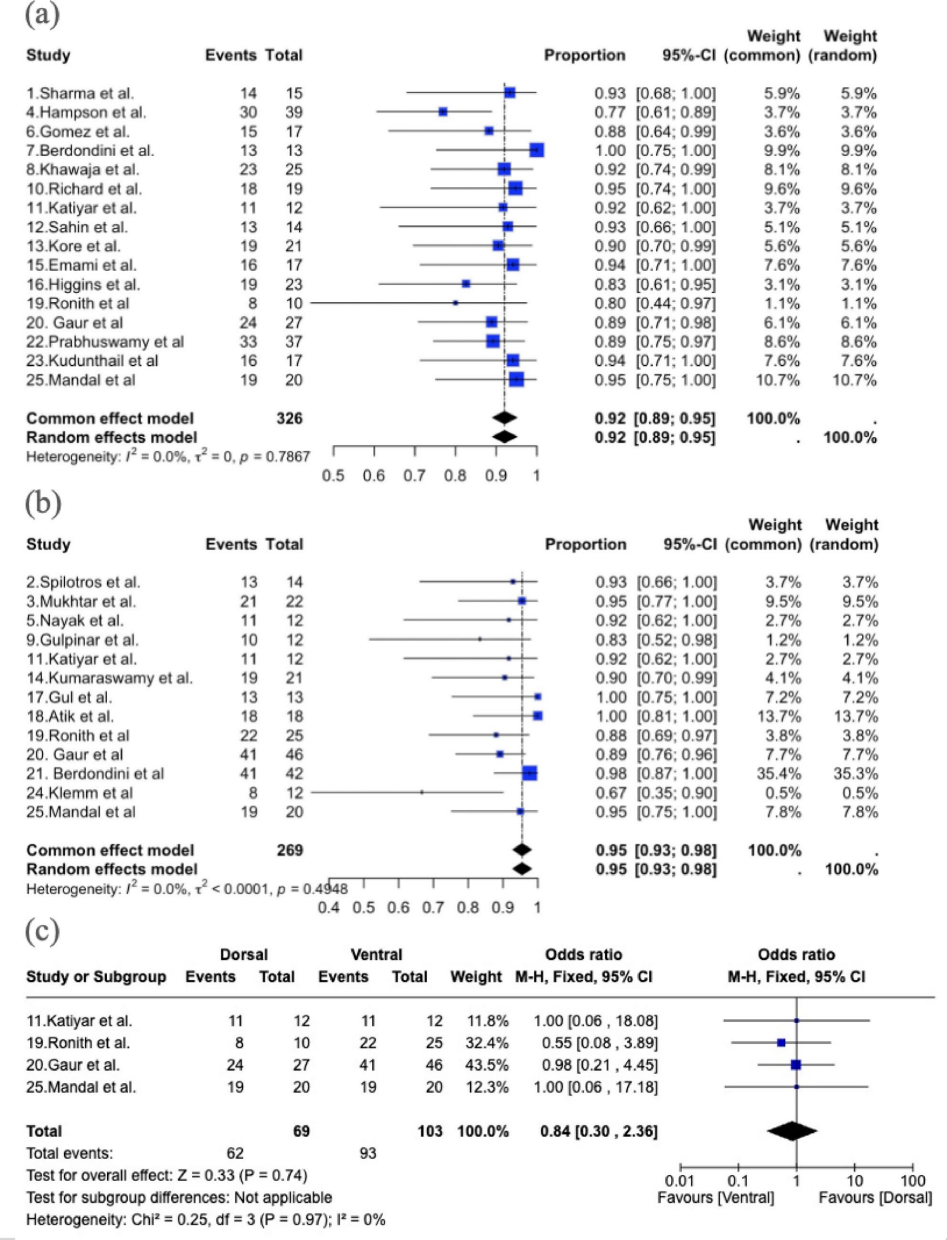
**a** Forest plot of success rates in urethroplasty using the dorsal technique. **b** Forest plot of success rates in urethroplasty using the ventral technique. **c** Forest plot of success rates in urethroplasty comparing dorsal and ventral technique

The first meta-analysis included four comparative cohort studies involving both dorsal and ventral patient groups. In the dorsal group, success was reported in 62 out of 69 patients, while in the ventral group, success was reported in 93 out of 103 patients. The overall OR for the success of the surgical technique, calculated using the Mantel-Haenszel method and a Fixed-Effect Model, was 0.84 (95% CI: 0.30–2.36; *p* = 0.74) (Fig. 2). The weighted median age was 42.3 years in the dorsal group and 44.4 years in the ventral group. The weighted median follow-up duration was 18.1 months in the dorsal group and 26.6 months in the ventral group. In all four studies, no complications were reported. This indicates no statistically significant difference between dorsal and ventral approaches regarding surgical success. Heterogeneity across the included studies was minimal, as demonstrated by a chi-squared (Chi²) value of 0.25 (df = 3; *p* = 0.97) and an I² statistic of 0%, suggesting consistency in the effect sizes reported by the studies.

The second and third meta-analyses evaluated the success rates of Dorsal and Ventral surgical techniques across 16 and 13 studies, respectively. For the Dorsal technique, the pooled success rate was 92.1% (95% CI: 89.1–95.1), with no significant heterogeneity among the studies (I² = 0.0%, τ² = 0, *p* = 0.79). Similarly, the Ventral technique demonstrated a pooled success rate of 95.5% (95% CI: 92.8–98.2), with low heterogeneity (I² = 0.0%, τ² < 0.0001, *p* = 0.49). (Fig. 2) Forest plots were generated to visualize the success rates and their confidence intervals for both techniques.

The Dorsal technique was successful in 291 out of 326 patients, while the Ventral technique was successful in 247 out of 269 patients. The test for heterogeneity indicated consistency in the effect sizes reported by the included studies, with I² = 0.0% for the Dorsal group and I² = 0.0% for the Ventral group, confirming low variability between studies.

**Fig. 3:**
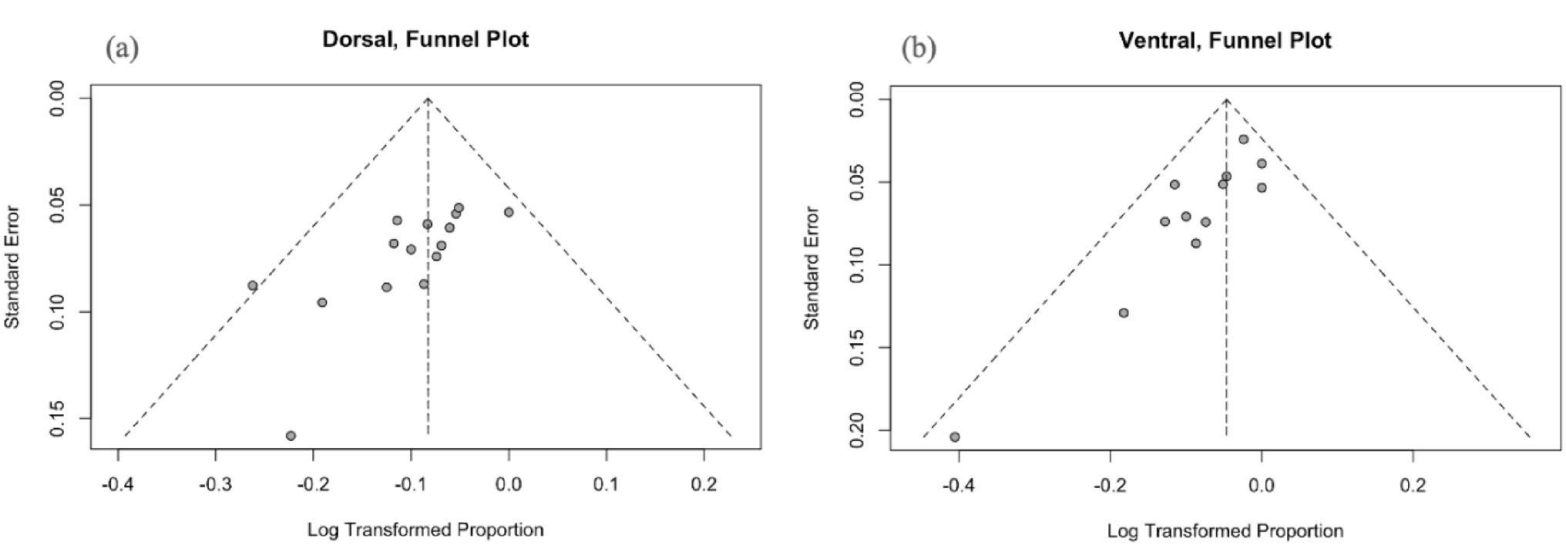
**a** Funnel plot of publication bias in dorsal technique. **b** Funnel plot of publication bias in ventral technique

The comparison of success rates between the two techniques using a Z-test yielded a Z-score of -1.6485 and a p-value of 0.099, indicating no statistically significant difference between the Dorsal and Ventral techniques (*p* > 0.05). The funnel plot for the dorsal technique showed no evidence of significant publication bias (Fig. 3a). However, the funnel plot for the ventral technique appeared asymmetrical, which may indicate potential publication bias or the presence of small-study effects (Fig. 3b).

Out of 25 studies, 21 were classified as level 4 evidence, reflecting single-arm observational designs with limited methodological rigor. Two studies were categorized as level 3, indicating retrospective non-randomized comparative designs. Only two studies met the criteria for level 2 evidence, both being randomized controlled trials (Supplementary Table 2).

## Discussion

With the increasing number of studies, the success of urethroplasty in the treatment of FUS has been well established. However, the choice between dorsal and ventral approaches remains debated. This meta-analysis, which included both comparative and pooled analyses, found no statistically significant difference in success rates between the dorsal and ventral approaches, with pooled success rates of 92.1% and 95.5%, respectively (*p* > 0.05). Minimal heterogeneity and the absence of publication bias further support the reliability of these findings. These results suggest that both approaches are effective, and decision-making should be guided by patient-specific factors and surgeon preference [11].

Over time, various urethroplasty techniques have been developed based on the type of graft or flap utilized, including vaginal, labial/vestibular, buccal, and lingual grafts, as well as vaginal and labial/vestibular flaps [13]. Among these, the buccal mucosal graft is the most widely adopted technique due to its superior outcomes, ease of harvest, minimal donor site morbidity, and favorable tissue characteristics for urethral reconstruction [19]. Another type of oral mucosa, the lingual mucosa, also offers favorable grafting properties, as the tongue can be easily manipulated within the oral cavity to facilitate graft harvesting [20]. Although Sharma et al. reported favorable outcomes (93%) in a cohort of 15 patients, the use of lingual mucosal grafts in female urethroplasty remains limited [15]. Lingual mucosal grafts have been used in only 2 of the 25 studies evaluating oral mucosal grafts in this meta-analysis (Table 1).

Despite the well-established efficacy of oral mucosal grafts in female urethroplasty, no consensus has been reached regarding the optimal approach—dorsal versus ventral—for graft placement. The lack of definitive guidelines underscores the need for further comparative studies to determine the most effective technique based on long-term outcomes and patient-specific factors.

The success of ventral buccal mucosal graft (BMG) urethroplasty in males is largely attributed to the abundant tissue in the bulbar urethra, which offers a well-vascularized and mechanically supportive environment for graft integration. A comparable supportive structure exists in the female urethra, where the periurethral fascia plays a crucial role in maintaining graft viability. While the preservation of this anatomical structure in ventral urethroplasty has been hypothesized to offer a potential advantage, our meta-analysis did not demonstrate a statistically significant difference in success rates between the ventral and dorsal approaches [21].

One of the most significant comparative studies in this field is the study by Gaur et al., which included 73 participants. Their findings demonstrated comparable success rates between the ventral (89.13%) and dorsal (88.89%) approaches, suggesting similar surgical outcomes. However, the study is subject to certain limitations, including its retrospective design, unequal comparison groups, and variations in follow-up duration, which may impact the reliability of its conclusions [22].

In addition to the surgical technique, various factors such as stricture length, location, number of dilations, and patient demographics can affect the success rate of urethroplasty [23]. In the study by Higgins et al., a single-center study on dorsal buccal mucosa urethroplasty, recurrence was reported in 4 out of 23 patients, with a median time to recurrence of 3.8 months. When comparing patients with and without recurrence in a subgroup analysis, those with stricture recurrence had a longer duration of symptoms and more dilations prior to urethroplasty. However, the small sample size is underpowered to find statistical significance [24]. In another study comparing the surgical characteristics of patients with and without recurrence, no preoperative factors significantly predicted recurrence, although those with recurrence had a slightly longer mean stricture length and smaller mean stricture caliber [25]. In the series by Kore et al., both patients who experienced failure developed urinary tract infections in the postoperative period [26]. Although patient characteristics are believed to influence recurrence, the limited number of patients with recurrence and the insufficient reporting of patient characteristics in many of the studies included in this meta-analysis have hindered the ability to conduct robust statistical analysis.

The ventral approach is hypothesized to offer several advantages, particularly in preserving neurovascular structures. By avoiding dorsal urethral dissection, this technique minimizes the risk of injury to the neurovascular structures of the clitoris, potentially reducing the likelihood of postoperative sexual dysfunction [27]. However, despite this theoretical benefit, Rohith et al. found no significant difference in Female Sexual Function Index (FSFI) scores between patients undergoing ventral urethroplasty (23.34 ± 8.73) and those undergoing dorsal urethroplasty (22.67 ± 11.06; *p* = 0.8) in their study of 25 ventral and 10 dorsal cases [28]. These findings suggest that the impact of urethroplasty on sexual function remains inconclusive, warranting further research with larger cohorts.

In the randomized controlled trial comparing 20 patients in each group, Mandal et al. observed a 95% success rate for both methods during a median follow-up of 21 months. The median operative time for the ventral approach was 30 (22–38) minutes, whereas the median time for the dorsal approach was 44.5 (40–52.5) minutes, which was statistically significantly longer (*p* < 0.005). The median blood loss (15 mL vs. 10 mL) was also statistically significantly greater in the dorsal approach. Furthermore, when comparing VAS pain scores, the ventral group exhibited a statistically significantly lower score during the first 24 h postoperatively. However, while these differences were statistically significant, their clinical relevance may be limited. The difference in blood loss (15 mL vs. 10 mL) is minimal and unlikely to have clinical impact. Similarly, the difference in VAS pain scores was approximately one point and was no longer present at 48 h postoperatively [29].

The three-dimensional U-shaped geometry of the female urethral sphincter results in a lower concentration of sphincter fibers at the ventral position (6 o’clock position). Consequently, the ventral urethrotomy approach is expected to better preserve sphincter integrity and reduce the risk of postoperative SUI [30]. In contrast, in this systematic review, a total of 4 cases of SUI complications were reported in two studies using the ventral approach, while 2 cases were reported in two studies using the dorsal approach. Despite ventral stricturotomy being performed, the study by Spilotros et al. reported the highest incidence of SUI, with 3 cases (3 out of 14 patients), all of which did not require further intervention and resolved with pelvic floor muscle training [1]. In another study presenting a patient series of urethroplasty using the ventral approach, SUI that developed in one patient also resolved with conservative measures within six months [31]. However, despite the theoretically increased risk of SUI in the dorsal approach due to the incision of the sphincter and pubo-urethral ligaments, the fact that only two out of 289 patients developed stress incontinence suggests that the dorsal technique remains a viable and safe option [24, 32]. These findings highlight the need for further research to better define the impact of each approach on post-urethroplasty continence outcomes.

One of the primary advantages of the dorsal approach is its ability to reduce the risk of vaginal injury and minimize the likelihood of urethrovaginal fistula formation [15]. This theoretical benefit is often cited as a key reason for preferring the dorsal technique in certain clinical scenarios. However, in this systematic review, none of the included studies reported the occurrence of urethrovaginal fistula, suggesting that the overall risk of this complication may be low regardless of the chosen surgical approach.

Aside from the complications previously mentioned, none of the studies reported grade 3 or higher complications according to the Clavien-Dindo classification [33]. The most frequently observed complication, urinary tract infection, occurred in 7 out of 39 patients (18%) in the case series by Hampson et al., which employed the dorsal approach [25]. In the study by Kore et al., urinary tract infections developed in two patients, both of whom also experienced recurrence [26]. In the ventral group, urinary tract infection, hemorrhage, and wound infection were each reported in one patient in the series by Gulpinar et al. [34]. In the study by Gomez et al., only one patient required a transfusion due to intraoperative bleeding, and it was noted that the patient’s preoperative stricture was radiation-induced [35]. Although some non-comparative studies have reported a higher number of complications in the dorsal group, the randomized controlled trials included in this review did not report any complications in either group. Therefore, the data do not support the conclusion that the ventral group has a lower complication rate. Additionally, due to heterogeneity in patient characteristics and study designs, a reliable comparison of complication rates between the two techniques cannot be made. A proper comparison would require well-designed randomized controlled trials that account for factors such as stricture length, stricture location, comorbidities, and patient age.

This meta-analysis has several limitations. The predominance of single-center and retrospective studies reduces the overall reliability and generalizability of the findings. Additionally, the comparative analysis is based on only four studies, with an unequal distribution of patients, further limiting the robustness of the results. The small sample size also restricts the ability to perform a comprehensive statistical analysis. Although recurrence of urethral stricture requiring intervention was consistently defined as treatment failure across all included studies, the variation in follow-up durations complicates a standardized comparison of success rates. Furthermore, objective measures of surgical success, such as Qmax, post-void residual volume (PMR), and AUA symptom scores, were not uniformly assessed across studies, limiting the scope of comparative analysis. Additionally, while the ventral approach is theoretically associated with a higher risk of sexual dysfunction, a more definitive evaluation would require the inclusion of validated sexual function questionnaires to strengthen the analysis of postoperative outcomes. The overall level of evidence is limited, as the majority of included studies were Level 4 observational designs, with only two randomized controlled trials meeting Level 2 criteria. This may reduce the strength and generalizability of the conclusions, highlighting the need for more rigorous, high-quality studies to confirm these findings.

The observed asymmetry in the funnel plot for the ventral technique may reflect underlying publication bias or small-study effects (Fig. 3b). This asymmetry suggests a potential publication bias or small-study effects that should be interpreted with caution. Such a pattern may result from selective reporting, underrepresentation of studies with negative or null findings, or greater heterogeneity in smaller studies. Although funnel plots serve as a valuable visual tool for detecting possible bias, they are not definitive and should be supported by further statistical analysis when drawing conclusions.

## Conclusion

This meta-analysis indicates that both dorsal and ventral approaches are effective for female urethral stricture repair, demonstrating high success rates. No statistically significant difference was found between the two techniques. Surgical choice should be guided by patient characteristics and surgeon expertise. Further multicenter, prospective studies with standardized outcome measures are needed to establish more definitive recommendations.

**Table 1:** Table summarizing all the studies included

Author	Year	Patients/*n*	Age/years	Previous interventions(*n*)	Follow-up/months	Graft	Technique	Postop evaluation	Succes rate	Complications (*n*)
1.Sharma et al. [15]	2009	15	42 (25–65) *	Dilatation & Urethrotomy (15)	12 **	LMG	Dorsal	VCUG, Uroflowmetry, Questionnaire	93%	Wound infection (1)
2.Spilotros et al. [1]	2016	14	-NA	-NA	12.1 *	BMG	Ventral	Uroflowmetry, PVR, resolution of voiding symptoms with no requirement for further urethral dilatations or intermittent self-catheterizations	93%	Stress incontinence (3)
3.Mukhtar et al. [31]	2017	22	50 (34–72) **	Dilatation (22)	21.5 (6–51) **	BMG	Ventral	Uroflowmetry (Qmax) PVR, Resolution of symptoms	95.5%	Stress incontinence (1)
4.Hampson et al. [25]	2019	39	50 (29–81) *	Dilatation (34)	33 (7–106) *	BMG	Dorsal	Uroflowmetry (Qmax) VCUG, cystoscopy	77%	Urinary tract infection (7)
5.Nayak et al. [36]	2019	12	41 (23–58) *	Dilatation (9)	18 (8–28) *	BMG	Ventral	Questionnaire: AUA-SS, Uroflowmetry, PVR	92%	N/A
6.Gomez et al. [35]	2020	17	51 (32–76) *	Dilatation (16)	15 (2–149) *	BMG	Dorsal	Patients requiring secondary procedure	87%	Intraoperative bleeding (1)
7.Berdondini et al. [37]	2021	13	56 (29–69) **	Dilatation (13) Urethroplasty (3)	11 (7–18) **	BMG	Dorsal	Patients requiring secondary procedure	100%	None
8.Khawaja et al. [38]	2021	25	46 (26–66) *	Dilatation (4)	25.5 (3–48) *	BMG	Dorsal	Uroflowmetry (Qmax) PVR	92%	None
9.Gulpinar et al. [34]	2021	12	56 (39–68) **	Dilatation (12) Urethroplasty (1)	25.5 (9–35) **	BMG	Ventral	Uroflowmetry, PVR	83.3%	Bleeding (1) Wound infection (1) Urinary tract infection (1)
10.Richard et al. [32]	2021	19	58 (+/- 13) ***	Dilatation (15) Urethroplasty (3)	12 (1–49) **	BMG/LMG	Dorsal	Questionnaire: PGI-I Uroflowmetry (Qmax) PVR	94.7%	Stress urinary incontinence (1) Sexual dysfunction (1)
11.Katiyar et al. [39]	2021	12	41.5 +-/ 10.4 *	Dilatation (21)	6 **	BMG	Dorsal	Questionnaire: AUA-SS Uroflowmetry (Qmax) PVR	91%	None
		12	49.3 +/- 10.9 *		6 **		Ventral		91%	None
12.Sahin et al. [40]	2022	14	47.5 (4–68) **	Dilatation (14)	12 **	BMG	Dorsal	Uroflowmetry (Qmax)	92.8%	None
13.Kore et al. [26]	2022	21	45 *	N/A	25 *	BMG	Dorsal	Questionnaire: AUA-SS Uroflowmetry (Qmax) PVR	90.5%	Urinary tract infection (2)
14.Kumaraswamy et al. [41]	2022	21	44 (28–59) **	Dilatation (15)	42 (24–64) **	BMG	Ventral	Questionnaire: AUA-SS Uroflowmetry (Qmax) PVR	90.5%	None
15.Emami et al. [42]	2023	17	54.8 (42–65) *	N/A	17.35 (12–23) *	BMG (lower lip)	Dorsal	Questionnaire: AUA Uroflowmetry (Qmax) PVR	94%	None
16.Higgins et al. [24]	2023	23	50 (34–84) **	Dilatation (20)	12.2 (1–81) **	BMG	Dorsal	Patients requiring secondary procedure, Uroflowmetry, PVR	83%	Stress incontinence (1) Urge incontinence (1)
17.Gul et al. [43]	2023	13	50 (44–62) **	Dilatation (12)	30 (12–30) **	BMG	Ventral	Questionnaire: AUA-SS, UDI, SF-36, FSFI Uroflowmetry, PVR	100%	None
18.Atik et al. [44]	2024	18	54 (35–78) **	Dilatation (18)	21.5 (1–35) **	BMG	Ventral	IPSS, Uroflowmetry, PVR,	100%	None
19.Ronith et al. [28]	2024	10	39 (33–50) **	Dilatation/ catheterization (6)	27 (12.5–48.75) **	BMG	Dorsal	Questionnaire: WHO-QOL, FSFI	80%	None
		25	43 (36–50) **	Dilatation/ catheterization (17)	35 (15.5–53) **		Ventral		88%	None
20.Gaur et al. [22]	2024	27	45 (37–52) **	Dilatation (3)/Surgery (5)	14 (7–17) **	BMG	Dorsal	Questionnaire: AUA-SS Uroflowmetry (Qmax) PVR	88.9%	None
		46	43 (38–50) **	Dilatation (19)/Surgery (3)	27.5 (11–55) **		Ventral		89.1%	None
21.Berdondini et al. [37]	2024	42	53.6 (23.8–72.2) *	Dilatation (42) Urethrotomy (6) Urethroplasty (2)	38.1 (16–75) *	BMG	Ventral	Uroflowmetry (Qmax) PVR	98%	None
22.Prabhuswamy et al. [45]	2024	37	47.8 (28–70) *	Dilatation (20)	30.2 (18–44) *	BMG	Dorsal	Uroflowmetry (Qmax) PVR	89%	ClavieneDindo Grade 1 complications in 6 out of 37 (16.2%) patients during hospital stay.
23.Kudunthail et al. [46]	2024	17	53.58 ± 17.63 *	Dilatation (6) Urethrotomy (1)	17.05 ± 31.43 *	BMG	Dorsal	Questionnaire: AUA Uroflowmetry (Qmax) PVR	94.1%	N/A
24.Klemm et al. [47]	2024	12	57 (48 − 67) **	Dilatation (7) Urethrotomy (10) Urethroplasty (3)	66 (18–91) **	BMG	Ventral	Uroflowmetry (Qmax) Questionnaire: ICIQ-FLUTS, PROM	66.6%	N/A
25. Mandal et al. [29]	2025	20	42 (36.5–51) **	Dilatation (8)	21 **	BMG	Dorsal	Questionnaire: AUA Uroflowmetry (Qmax) PVR	95%	None
		20	48.5 (38–52) **	Dilatation (12)		BMG	Ventral		95%	None

*BMG* Buccal mucosal graft, *LMG* Lingual mucosal graft, *N/A* Non available, *PVR* Post-void residual, *AUA-SS* American urological association symptom score, *UDI* Urinary distress inventory, *SF-36* Short form-36 health survey, *FSFI* Female sexual function index, *IPSS* International prostate symptom score, *Qmax* Maximum flow rate, *VCUG* Voiding cystourethrography, *PGI-I* Patient global impression of improvement, *WHO-QOL* World health organization quality of life, *ICIQ-FLUTS* International consultation on incontinence questionnaire - female lower urinary tract symptoms, *PROM* Patient-reported outcome measures

*mean [48]

**median [48]

***mean (standard deviation).

## Electronic supplementary material

Below is the link to the electronic supplementary material.

Supplementary Material 1

## Data Availability

The list of articles found before screening and the raw tables with extracted data are available upon appropriate request to the corresponding author.
