# Supplementary material for: Comparing ventral and dorsal oral mucosal graft urethroplasty in female urethral stricture: a systematic review and meta-analysis
Source: World J Urol. 2025 Jun 26;43(1):397. doi: 10.1007/s00345-025-05773-4 (PMC12202565; doi:10.1007/s00345-025-05773-4)
Supplement: Supplementary file 1 — Supplementary Material 1 [file 345_2025_5773_MOESM1_ESM.docx]

**Comparing ventral and dorsal oral mucosal graft urethroplasty in female urethral stricture: A systematic review and meta-analysis**

Mazhar Ortac^1^, M. Firat Ozervarli^1^, Rifat Burak Ergul^1^, Arda Tunc Aydinoglu^1^, Mevlut Melih Bicer^1^, Teresa Olsen Ekerhult^2^, Senol Tonyali^1^

1. Department of Urology, Istanbul University, Istanbul Faculty of Medicine, Istanbul, Turkey
2. Department of Urology, Institute of Clinical Sciences, Sahlgrenska Academy, University of Gothenburg, Gothenburg, Sweden

**Corresponding author:**

Mazhar Ortac, Assoc Prof

Adress: Topkapı, Turgut Özal Millet Street, 34093 Fatih/İstanbul

E-mail: mazhar.ortac@istanbul.edu.tr

Tel: +90 505 550 06 18

Fax: +90 212 4142000

Orcid ID: 0000-0002-4784-5209

**Content: Supplementary Tables**

**Supplementary Table 1:** Search strings

| **MEDLINE** | ((urethroplasty[Title/Abstract]) OR (urethral stricture[Title/Abstract])) AND ((female[Title/Abstract]) OR (women[Title/Abstract])) AND ((buccal[Title/Abstract]) OR (ventral[Title/Abstract]) OR (lingual[Title/Abstract]) OR (mucosal[Title/Abstract])) |
| --- | --- |
| **Scopus** | ( TITLE-ABS-KEY ( urethroplasty ) OR TITLE-ABS-KEY ( urethral AND stricture ) AND TITLE-ABS-KEY (female ) OR TITLE-ABS-KEY ( women ) AND TITLE-ABS-KEY ( buccal ) OR TITLE-ABS-KEY ( ventral ) ORTITLE-ABS-KEY ( lingual ) OR TITLE-ABS-KEY ( dorsal ) ) |
| **Web of Science Core Collection** | **ALL=(("urethroplasty" OR "urethral stricture" OR "urethral disease") AND ("female" OR "women") AND ("buccal" OR "lingual" OR "ventral" OR "dorsal" OR "ventral inlay" OR "ventral onlay" OR "dorsal onlay"))** |

**Supplementary Table 2:** Quality assessment table

| **Author** | **Study design** | **Study quality/Risk of bias*****  *Total score* | **Level of Evidence****** |
| --- | --- | --- | --- |
| 1.Sharma et al. | Prospective, single arm | 7^b^ | 4 |
| 2.Spilotros et al. | Retrospective, comparatative | 5^b^ | 4 |
| 3.Mukhtar et al. | Prospective, single arm | 9^b^ | 4 |
| 4.Hampson et al. | Retrospective, multi-center, single arm | 9^b^ | 4 |
| 5.Nayak et al. | Retrospective, single arm | 7^b^ | 4 |
| 6.Gomez et al. | Retrospective, single arm | 7^b^ | 4 |
| 7.Berdondini et al. | Retrospective, single arm | 7^b^ | 4 |
| 8.Khawaja et al. | Prospective, single arm | 7^b^ | 4 |
| 9.Gulpinar et al. | Prospective, single arm | 8^b^ | 4 |
| 10.Richard et al. | Retrospective, single arm | 8^b^ | 4 |
| 11.Katiyar et al. | Prospective, randomised controlled study | 2^c^ | 2 |
| 12.Sahin et al. | Retrospective, single arm | 8^b^ | 4 |
| 13.Kore et al. | Retrospective, single arm | 9^b^ | 4 |
| 14.Kumaraswamy et al. | Retrospective, single arm | 8^b^ | 4 |
| 15.Emami et al. | Prospective, single arm | 9^b^ | 4 |
| 16.Higgins et al. | Retrospective, single arm | 10^b^ | 4 |
| 17.Gul et al. | Retrospective, single arm | 7^b^ | 4 |
| 18.Atik et al. | Retrospective, single arm | 8^b^ | 4 |
| 19.Ronith et al | Retrospective, non randomized | 6^a^ | 3 |
| 20. Gaur et al | Retrospective, non randomized | 7^a^ | 3 |
| 21. Berdondini et al | Retrospective, single arm | 8^b^ | 4 |
| 22. Prabhuswamy et al | Retrospective, single arm | 7^b^ | 4 |
| 23. Kudunthail et al | Retrospective, single arm | 8^b^ | 4 |
| 24. Klemm et al | Retrospective, single arm | 8^b^ | 4 |
| 25. Mandal et al | Prospective, randomised controlled study | 3^c^ | 2 |

***According to Newcastle-Ottawa Scale for cohort studies (range: 0-9) or JBI's tool for case series (range: 0-10) or Jadad Scale for randomized studies (range: 0-5); **** According to Oxford Centre for Evidence-Based Medicine 2011 (range: 1-5)

^a^: Newcastle-Ottawa Score; ^b^: JBI Score; ^c^: Jadad Score
